# Enhancing Calprotectin’s Predictive Power as a Biomarker of Endoscopic Activity in Ulcerative Colitis: A Machine Learning Use Case

**DOI:** 10.3390/biomedicines12030475

**Published:** 2024-02-20

**Authors:** Mihaela Dranga, Cătălina Mihai, Otilia Gavrilescu, Cristina Cijevschi Prelipcean, Iolanda Valentina Popa

**Affiliations:** 1 Internal Medicine Department, “Grigore T. Popa” University of Medicine and Pharmacy, 700115 Iasi, Romania; mihaela_dra@yahoo.com (M.D.); iolivp@gmail.com (I.V.P.); 2 “Saint Spiridon” County Hospital, 700111 Iasi, Romania; cristinacijevschi@yahoo.com

**Keywords:** ulcerative colitis, calprotectin, endoscopic disease activity, non-invasive biomarkers, machine learning

## Abstract

Ulcerative colitis (UC) is a chronic inflammatory bowel disease characterized by periods of exacerbation and remission, making disease monitoring and management challenging. Endoscopy, the gold standard for assessing disease activity and severity, involves invasive procedures and is associated with patient discomfort and risks. Using machine learning (ML) to combine fecal calprotectin with other clinical or biological tests can significantly enhance the non-invasive prediction of endoscopic disease activity (EDA) in UC. **Aim:** To prove that by fusing fecal calprotectin with other clinical data into an ML model, the performance of the non-invasive prediction of EDA can be significantly improved. **Methods:** We conducted a prospective, observational, single-center study encompassing 103 patients diagnosed with UC. We employed multilayer perceptron models as the core ML algorithm for predicting EDA. For the constructed models, we utilized the varImp function from the caret library in R to assess the significance of each variable in predicting the outcome. **Results:** Calprotectin as a sole predictor obtained an accuracy of 70% and an area under the curve (AUC) of 0.68. Combining calprotectin with the list of selected predictors that were fed to the MLP models improved accuracy and the AUC. The accuracy of the algorithm on the test set was 85%. Similarly, the AUC increased to 0.93. This is the first study to propose the use of calprotectin as a predictor in an ML model to estimate UC endoscopic disease activity. **Conclusion**: The deployment of this ML model can furnish doctors and patients with valuable evaluation of endoscopic disease activity which can be highly beneficial for individuals with UC who need long-term treatment.

## 1. Introduction

Ulcerative colitis (UC) is a chronic inflammatory bowel disease (IBD) characterized by inflammation of the colonic mucosa. The disease is characterized by periods of exacerbation and remission, making disease monitoring and management challenging.

Endoscopy, the gold standard for assessing disease activity and severity, involves invasive procedures and is associated with patient discomfort and risks [1]. As a result, there has been growing interest in identifying non-invasive biomarkers that can accurately reflect endoscopic disease activity (EDA) in UC [2].

There is a collective body of research aiming to elucidate the pathogenesis of inflammation in IBD [3,4,5] and potential biomarkers to predict inflammatory status [6,7,8]. This supports the use of biomarkers like calprotectin for disease monitoring as a substitute for endoscopy in certain clinical scenarios by exploring the relevance of fecal biomarkers in reflecting mucosal healing (a key aspect in the control of IBD) [9]. The current literature also emphasizes precision medicine approaches in IBD treatment, highlighting the importance of personalized therapeutic strategies [10].

Fecal calprotectin has been studied as a sole biomarker in UC with good results [11]; however, it leaves room for improvement in terms of performance metrics.

Using machine learning (ML) to combine fecal calprotectin with other clinical or biological tests can significantly enhance the non-invasive prediction of endoscopic disease activity in UC for several reasons. Fecal calprotectin and other clinical tests provide different types of information about the inflammatory status of the gut [12]. Fecal calprotectin reflects local inflammation in the intestines, while blood tests can capture systemic markers of inflammation. Combining these sources of information can offer a more comprehensive and accurate picture of disease activity. Moreover, ML models excel at handling multimodal data [13,14,15,16,17], such as combining fecal calprotectin with clinical and blood biomarkers. By effectively integrating diverse data types, these models can capture complex relationships that might not be evident when analyzing each biomarker separately. Combining multiple biomarkers through ML techniques often leads to improved predictive accuracy [18].

Our study hypothesizes that the integration of fecal calprotectin levels with additional clinical and biological markers into an ML framework can improve the prediction of EDA in patients with UC. In light of the limitations associated with current non-invasive biomarkers when used in isolation, we seek to investigate whether an ML-based model that merges multiple biomarkers can provide a more accurate and reliable non-invasive assessment of EDA in UC. This study seeks to establish the potential of ML algorithms in enhancing diagnostic precision beyond the existing capabilities of individual biomarkers.

We aim to prove that by fusing fecal calprotectin with other clinical data into an ML model, the performance of the non-invasive prediction of EDA can be significantly improved. By leveraging the strengths of the biomarkers studied so far and the capabilities of ML algorithms, clinicians can achieve more accurate, personalized, and timely assessments of disease activity, ultimately improving patient care and management.

## 2. Materials and Methods

### 2.1. Study Design

We conducted a prospective, observational, single-center study encompassing 103 patients diagnosed with UC. Patients were admitted to the hospital from January 2012 to December 2022. The diagnosis of UC was established through comprehensive hematological and biochemical assessments as well as endoscopic and imaging examinations alongside histopathological confirmation according to the European guidelines in force [19].

All study participants fulfilled the following inclusion criteria: (1) patients diagnosed with ulcerative colitis (UC) as per the European Crohn’s and Colitis Organisation (ECCO) guidelines; (2) age of 18 years and older; (3) patients who underwent a complete colonoscopy and were scored using the Mayo endoscopic score; (4) patients who completed a semiquantitative calprotectin stool test along with the colonoscopy; (5) patients able and willing to provide written informed consent for participation in this study.

Exclusion criteria were defined as follows: (1) patients younger than 18 years; (2) individuals with infections or autoimmune and inflammatory disorders other than UC which could independently affect inflammatory biomarkers; (3) patients with a history of colorectal surgery or any other condition likely to interfere with calprotectin levels; (4) patients with diagnosed cirrhosis, neoplasms, or those undergoing hemodialysis, to avoid confounding physiological or pathological factors; (5) patients with incomplete data records which did not allow for a full assessment of all the parameters required for this study; (6) patients unwilling or unable to consent to the study procedures.

Individuals with concurrent conditions, including infections, autoimmune and inflammatory disorders, cirrhosis, neoplasms, or undergoing hemodialysis, were not considered in this study as these factors could potentially impact medical measurements and outcomes and were therefore excluded.

This study was approved by the local Ethics Committee at “Grigore T. Popa” University of Medicine and Pharmacy, Iasi, Romania (No. 125/04.10.2019). Each patient provided written informed consent.

### 2.2. Data Acquisition

For all patients, the full UC disease activity index (Mayo score) was measured [20]. We defined the disease activity index based on clinical symptoms (DAIS) as the sum of the stool frequency and rectal bleeding subscores (the first two items from the full Mayo score).

This study involved gathering various continuous variables as part of the collected parameters: age, body mass index—BMI, smoking—pack years, total proteins, hemoglobin, mean corpuscular volume—MCV, mean corpuscular hemoglobin concentration—MCHC, platelets, white blood cells—WBC, erythrocyte sedimentation rate—ESR, fibrinogen, C reactive protein—CRP, DAIS. Several categorical variables were also collected: sex, Mayo endoscopic score, calprotectin, and disease extent.

Disease extent in UC is classified as proctitis (E1), left-sided (E2), or extensive colitis (E3).

Calprotectin was measured using a semiquantitative rapid test. CalDetect^®^ Sofar SpA, Trezzano. Rosa, Milan, Italy is based on a chromatographic immunoassay technique. It detects the following three ranges of concentration of calprotectin in feces: <15, 15–60, and >60 μg/g. Concentrations lower than 15 μg/g were labeled with a value of 1. Concentrations between 15 and 60 μg/g were marked with a value of 2. Similarly, a label of 3 was set for calprotectin concentrations over 60 μg/g.

Colonoscopies were performed by physicians from the Gastroenterology and Hepatology Institute in Iași, Romania, utilizing the EVIS EXERA II endoscopy system (Olympus America, Westborough, MA, USA). The assessment of the endoscopic Mayo score followed the most recent European consensus guidelines [19]. A score of 0 or 1 on the endoscopic Mayo scale defines endoscopic remission, while a score of 2 or 3 indicates the presence of active disease.

### 2.3. Outcome Definition

The outcome to be predicted by our models was a binary variable that estimates whether the patient has active endoscopic disease or remission according to the Mayo binary classification defined above.

### 2.4. Preprocessing

Continuous variables that were documented underwent normalization within the [0, 1] range. Hemoglobin and hematocrit values were adjusted to account for gender-based variations.

Instances with incomplete data were excluded, leading to a final database containing a total of 98 records.

All the calculations in this study were performed in R Studio (version 1.4.1106).

### 2.5. Calprotectin as a Unique Disease Activity Biomarker

Initially, we employed the pROC::roc and pROC::coords functions in R to determine the predictive power of calprotectin as a single attribute to estimate EDA. Given a predictor and a binary outcome, the pROC::roc function builds a receiver operating characteristic curve (ROC), computes the area under the ROC (AUC) and the confidence interval (CI), and plots the curve if requested. The pROC::coords function returns the coordinates of the ROC curve, such as accuracy, AUC, sensitivity, specificity, and positive (PPV) and negative predictive values (NPV), for an optimal threshold that maximizes Youden’s index.

### 2.6. Feature Selection

Due to the non-normal distribution of continuous parameters, we employed the Kruskal–Wallis rank sum test to determine the correlation between the collected continuous variables and the specified outcome.

To investigate potential associations between each categorical parameter and endoscopic activity, we conducted Pearson’s chi-squared test of independence.

The continuous and categorical parameters that exhibited correlation with the outcome (*p* < 0.1), as established by the Kruskal–Wallis and Pearson’s chi-squared tests, were chosen as predictive features for the machine learning models.

### 2.7. Development of the ML Models

We performed a random partition of the initial dataset comprising 98 UC patients, allocating 80% (78 records) to the training set and 20% (20 records) to the test set.

We employed multilayer perceptron (MLP) models as the core machine learning algorithm for predicting endoscopic disease activity (EDA). MLPs are a class of feedforward artificial neural networks that consist of at least three layers of nodes: an input layer, a hidden layer, and an output layer. We chose MLPs for their ability to capture complex non-linear relationships between input features and the target variable.

During model validation, we applied 10-fold cross-validation, repeating this process 10 times to ensure that our findings are robust and not due to random chance within the training data. This validation approach strengthened the generalizability of our results. Moreover, only the training set was used for training and developing the MLP models. The test set did not participate in the training stage and was used only to assess the models’ performance on new, unseen data.

We also recognize potential limitations in our study design, such as sample size and the single-center nature of this study, which could influence the diversity of the dataset and the external validity of our model. To contend with these limitations in future research, we suggest multicenter studies with larger patient cohorts, enabling the capture of a more heterogeneous patient population and further refining the predictive accuracy of our ML approach.

We created two MLP classifiers using the caret::train function within R Studio for model development. To ensure the reproducibility of this study, the parameters used in the caret::train function are detailed in Table 1.

The first MLP model was developed to predict EDA based on the selected variables, excluding calprotectin. The second MLP classifier was built to estimate EDA based on all the selected predictors, including the calprotectin score.

We assessed the classification accuracy of the ML models on both the test and training sets. Moreover, we calculated the AUC, sensitivity, specificity, as well as PPV and NPV.

### 2.8. Variable Importance

For both constructed models, we utilized the varImp function from the caret library in R to assess the significance of each variable in predicting the outcome. In the case of MLP models, the varImp function accepts the resultant MLP model as an argument and ranks variable importance using a ‘filter’ approach (ROC curve analysis).

## 3. Results

This study included 98 UC patients (after excluding records with missing values), of whom 67 (68.4%) were male and 31 (31.6%) were female. The ages of the participants ranged from 20 to 75 years.

Table 2 provides an overview of the selected parameters for all patients and within each endoscopic activity class. Continuous variables are presented as medians (interquartile ranges), while categorical variables are displayed as the frequency of occurrences for each category.

**Figure 1:**
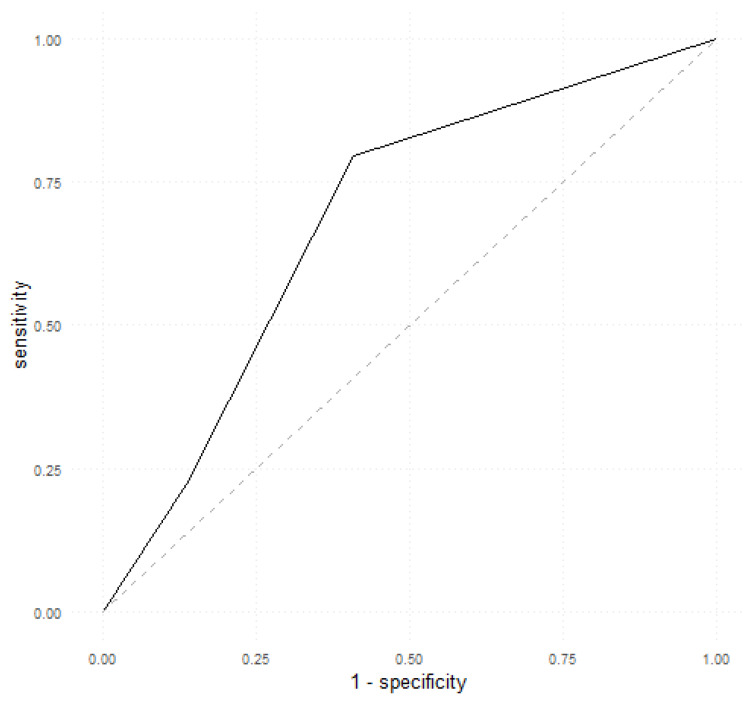
ROC curve depicting calprotectin as a single predictor of EDA.

Firstly, we assessed the performance of calprotectin as a single predictor of EDA using the pROC::roc function. The optimal threshold was 1.5. Therefore, a value of 1 (when assessing fecal calprotectin using a semiquantitative test) predicted an endoscopic remission, whilst values of 2 and 3 predicted an endoscopic relapse. The performance metrics are shown in Table 3 and the ROC curve is illustrated in Figure 1. Calprotectin as a sole predictor obtained an accuracy of 70% (95% CI, 0.6–0.78), a sensitivity of 59%, a specificity of 79.63%, and an AUC of 0.6848.

Next, the steps for building the ML models follow.

Prior to model development, the feature selection step was carried out. The Kruskal–Wallis rank sum (Table 4) and chi-square (Table 5) tests were applied to identify the continuous and categorical variables that are significantly related to endoscopic Mayo scores.

The feature selection step identified the following predictors to be used for the training of the ML models (*p* < 0.1): DAIS, platelet count, fibrinogen, and calprotectin.

Based on the variables selected as predictors by the feature selection step, two ML models were trained.

We developed the first MLP model to predict EDA based on the selected variables, excluding calprotectin. Table 6 presents a comparative overview of the performance metrics achieved by these classifiers. On the train set, the first model obtained an accuracy of 79.49% (95% CI, 0.69–0.88; *p* < 0.001) with a sensitivity of 69.7%, a specificity of 86.67%, a PPV of 79.31%, an NPV of 79.59% and an AUC of 0.86. On the test set, the model achieved a good performance with an accuracy of 80% (95% CI, 0.56–0.94; *p* = 0.01886), a sensitivity of 90.91%, a specificity of 66.7%, a PPV of 76.92%, an NPV of 85.71% and an AUC of 0.9192.

**Figure 2:**
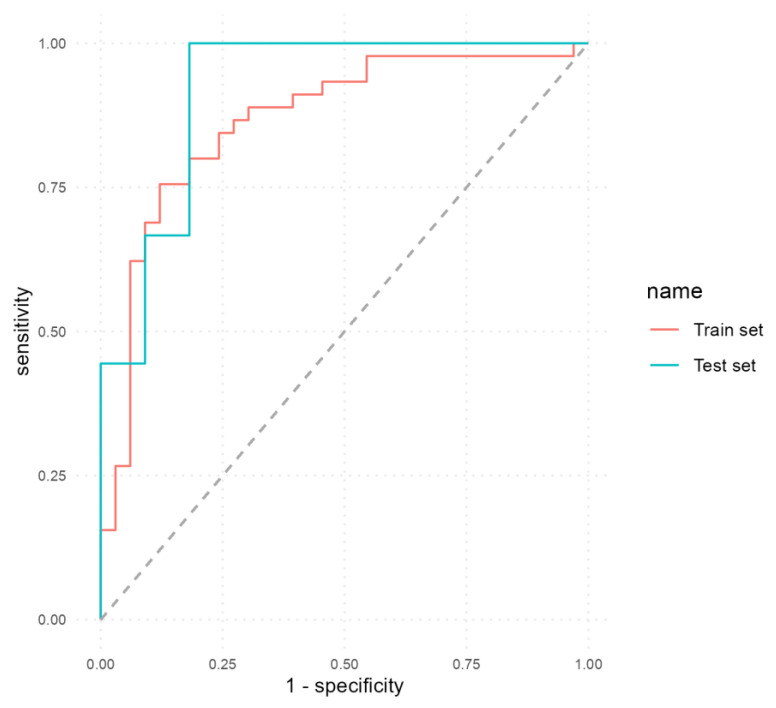
ROC curves of the first MLP model that excluded calprotectin as a predictor.

Figure 2 illustrates the ROC curves obtained by the first MLP model on both the train and test sets.

We developed a second MLP classifier to predict EDA using all the selected predictors, including calprotectin. The performance metrics attained by this classifier are detailed in Table 7. In the train and test sets, the model achieved a good performance: accuracy 86% (95% CI, 0.76–0.93; *p* < 0.001), sensitivity 78.12%, specificity 91.3%, PPV 86.21%, NPV 85.71%, AUC 0.88 (train set); and accuracy 85% (95% CI, 0.62–0.97; *p* = 0.016), sensitivity 83.33%, specificity 87.5%, PPV 90.91%, NPV 77.78%, AUC 0.93 (test set).

**Figure 3:**
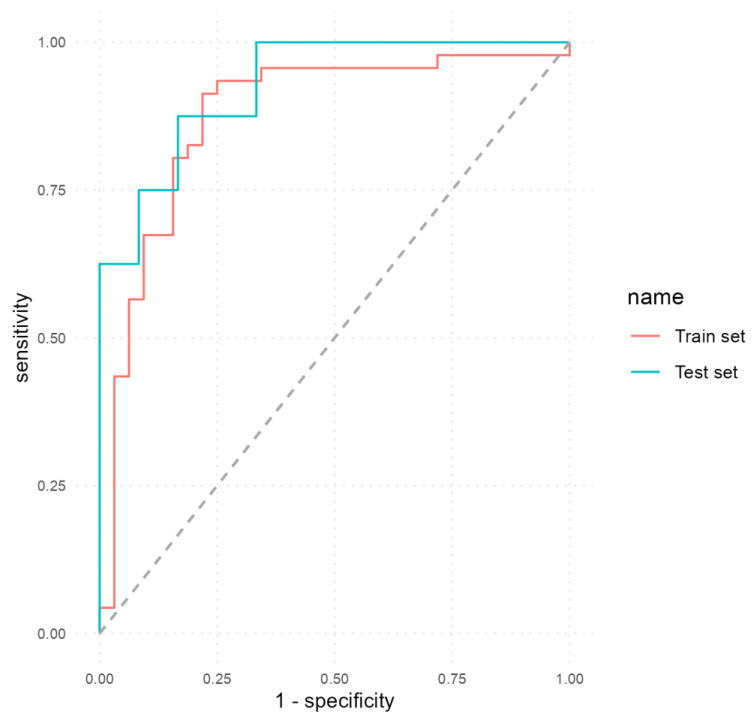
ROC curves of the second MLP model that included calprotectin as a predictor.

Figure 3 shows the ROC curves that illustrate the performance of the second MLP model on both the train and test sets. It can be seen how the inclusion of calprotectin as a predictor determined an increase in all performance metrics.

**Figure 4:**
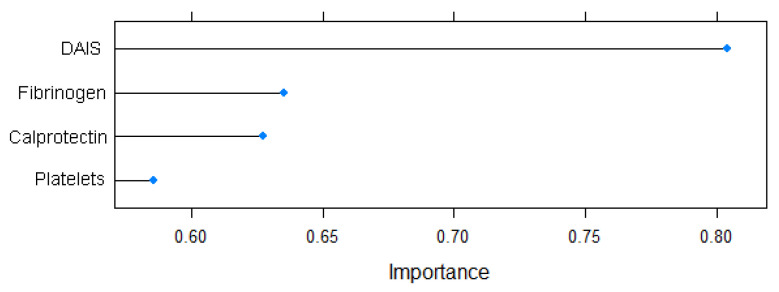
Variable importance for the EDA prediction model.

Variable importance as determined by the varImp function is graphically represented in Figure 4.

## 4. Discussion

Our research proposed an ML method that improves calprotectin’s predictive power as a biomarker of EDA in UC patients using low-cost laboratory data. This is the first study to propose the use of an ML model to estimate UC EDA that combines calprotectin with several routine laboratory data. Our findings indicate that it is possible to distinguish between active and inactive UC with high accuracy using non-invasive biological predictors.

Calprotectin, a calcium-binding protein found predominantly in neutrophils, has emerged as a promising biomarker of EDA in IBD. Also known as S100A8/A9 or myeloid-related protein (MRP) 8/14, calprotectin is a heterodimeric complex composed of S100A8 and S100A9 proteins. It is released into the extracellular environment upon neutrophil activation and is involved in various immune responses and inflammatory processes. Calprotectin functions by binding to divalent cations like calcium and zinc, thereby modulating the immune response and promoting neutrophil chemotaxis and adhesion to endothelial cells. Due to its role in inflammation, calprotectin levels have been investigated as a potential marker of disease activity in several inflammatory conditions, including IBD [21].

Multiple studies have reported a strong association between fecal calprotectin levels and endoscopic disease activity in UC [22,23].

While fecal calprotectin has shown promising results as a biomarker for assessing endoscopic disease activity in UC, there is always room for improvement in terms of performance metrics. Calprotectin’s performance as a biomarker is considered good but not without limitations. Fecal calprotectin levels can vary due to factors such as diet, medication use, and infections [24]. This variability can impact the reliability of the biomarker’s results. Moreover, determining appropriate cutoff values for distinguishing between active and inactive disease is important. The optimal cutoff can vary among different patient populations and may need adjustment based on individual characteristics [25]. Therefore, combining calprotectin with other biomarkers or clinical parameters could potentially enhance its diagnostic accuracy and predictive value.

Initially, this study underwent a feature selection process and identified a range of non-invasive predictors associated with EDA. These predictors included DAIS, platelet count, fibrinogen, and calprotectin. This alignment with the existing scientific literature is consistent, as all of these variables have been extensively explored in the pursuit of an optimal biomarker for evaluating EDA [26,27,28]. Nevertheless, our paper stands out by being a pioneer in incorporating calprotectin alongside other routine biological variables into a reliable predictor.

Subsequently, we utilized two MLP algorithms and conducted a comparative analysis of their performance. The results show that the inclusion of other biomarkers alongside calprotectin in the list of predictors that were fed to the models improved all performance metrics. As such, combining calprotectin and other biomarkers performs better in estimating endoscopic disease activity than calprotectin or clinical data alone.

The ROC curves generated in our study depict a trade-off between sensitivity and specificity in predicting endoscopic disease activity (EDA) in patients with UC. A higher AUC indicates better discriminatory power of the model, with an AUC of 1 indicating perfect discrimination between active disease and remission. In our study, calprotectin as a sole predictor obtained an AUC of 0.68 and an accuracy of 70% while MLP models achieved AUC values of 0.91 (first model) and 0.93 (second model) and accuracy values of 80% (first model) and 85% (second model). These findings suggest that the inclusion of calprotectin, combined with other clinical and biological parameters, substantially enhances the accuracy of non-invasive prediction of endoscopic disease activity in UC patients.

From a clinical perspective, the improved accuracy provided by our ML models holds promising implications for disease management. Accurate prediction of EDA can help clinicians identify patients who are more likely to require intensified therapy or closer monitoring to prevent disease progression and long-term complications. Additionally, the integration of multiple biomarkers, such as fecal calprotectin and clinical variables, offers a more comprehensive assessment of EDA, potentially reducing the need for invasive endoscopic procedures and associated risks and discomfort for patients. The deployment of such ML models may contribute to more personalized and timely interventions, ultimately enhancing patient care and optimizing treatment strategies in the management of ulcerative colitis.

While several studies have examined the predictive power of individual biomarkers, including calprotectin, we proved that the integration of multiple biomarkers can provide a more comprehensive and accurate assessment of disease activity. To further support our argument, we reviewed the existing literature on calprotectin as a biomarker for UC. We found several studies that solely focused on calprotectin and its predictive capabilities in assessing endoscopic disease activity in UC patients. Many publications demonstrated that fecal calprotectin levels correlated well with endoscopic activity in UC patients [29,30]. Similarly, the study conducted by D’Haens showed a strong association between calprotectin levels and endoscopic inflammation, suggesting its potential as a reliable biomarker [31]. Moreover, Sonoyama et al. compared different non-invasive biomarkers for disease activity assessment in UC and concluded that calprotectin had superior predictive power [32]. The findings from a meta-analysis which looked at the effectiveness of fecal calprotectin in assessing EDA in UC showed that it had a sensitivity of 87.3% (with a confidence interval of 85.4% to 89.1%), a specificity of 77.1% (with a confidence interval of 73.7% to 80.3%), and an AUC of 0.91 [33]. In our study, the ML approach yielded even higher specificity and AUC values compared to those reported in the meta-analysis. What needs to be emphasized is that we obtained these results using semi-quantitative tests for measuring fecal calprotectin. It is expected that in future studies, where we intend to use quantitative tests, the results will be even better.

While these studies highlight the significance of calprotectin as a standalone biomarker, our study goes beyond that by integrating calprotectin with other clinical and biological variables using ML techniques. By leveraging the strengths of these multimodal data inputs, our approach offers enhanced predictive accuracy and a more personalized assessment of disease activity in UC patients.

To date, there is only one study that combined calprotectin with other clinical data with the aim of predicting endoscopic activity in IBD. Guez et al. formulated a multimodal random forest framework that combined magnetic resonance enterography (MRE) information with biochemical indicators, including fecal calprotectin [34]. Their model demonstrated superior predictive capabilities for Crohn’s disease compared to a linear regression model relying solely on the Magnetic Resonance Index of Activity. However, MRE is an expensive and not immediately available technique. Conversely, our research is based only on inexpensive, readily available clinical parameters.

### Limitations

The first limitation lies in the small size of our dataset, and furthermore, the independent test set originates from the same center. This indicates the necessity, moving forward, to engage in extensive external validation using data obtained from various other centers. The second issue is the use of semiquantitative calprotectin stool tests. Our future studies will consider employing full quantitative fecal calprotectin tests for improved performance.

## 5. Conclusions

This study highlights how ML models have the capacity to integrate together diverse data modalities such as fecal calprotectin and other clinical data, leading to improved predictive accuracy of EDA, surpassing what can be achieved through traditional statistical methods. Advancements in refined ML techniques hold the potential to aid in the identification of personalized treatment plans and follow-up strategies for UC patients. By leveraging the strengths of ML techniques, we have made significant strides in the non-invasive prediction of EDA, ultimately enhancing disease monitoring and treatment management for UC patients. In our future studies, it is crucial to engage in extensive external validation using data obtained from various other centers to ensure the robustness and reliability of our ML models. This future direction will expand the applicability and impact of our research in a real-world clinical setting. Our future investigations will also incorporate full quantitative fecal calprotectin tests, which offer more precise quantification, thereby further improving the performance of our models.

## Figures and Tables

**Table 1:** Parameters employed for training the ML models that were passed on to the caret::train function.

method	“mlpML”
tuneLength	5
preProcess	c(“scale”, “center”)
trControl	trainControl(method = “repeatedcv”, number = 10, repeats = 10, sampling = “smote”)

**Table 2:** Gender, age, and the selected parameters for all patients and each endoscopic activity class, respectively.

	All	Endoscopic Remission	Endoscopic Relapse
Number of records	98	44	54
Gender (male/female)	67:31	28:16	39:15
Age (years)	46 (22)	46 (22)	46.5 (21.75)
DAIS	4 (3)	2 (3)	5 (3)
Fibrinogen	353 (112.5)	329 (82.5)	396 (158.5)
Platelets	298,000 (127,250)	270,500 (116,000)	301,000 (119,250)
Calprotectin categories (1:2:3)	37:43:18	26:12:6	6:19:11

DAIS (disease activity index based on clinical symptoms).

**Table 3:** Performance metrics of calprotectin as a sole predictor of EDA.

	Actual Values	
Predicted Values	Remission	Activity
Remission	26	11
Activity	18	43
Accuracy	0.7041	
95% CI	(0.6034, 0.7921)	
Sensitivity	0.5909	
Specificity	0.7963	
PPV	0.7027	
NPV	0.7049	
AUC	0.6848	

CI (confidence interval); PPV (positive predictive value); NPV (negative predictive value); AUC (area under the curve).

**Table 4:** Outcomes obtained from utilizing the Kruskal–Wallis method to identify the continuous variables with significant associations to the EDA.

Parameter	Chi-Square	df	*p*
Age	1.1414	3	0.7671
BMI	1.6682	3	0.644
Smoking—pack years	5.5194	3	0.1375
Total proteins	3.6729	3	0.299
Hemoglobin	1.7151	3	0.6336
MCV	5.635	3	0.1308
MCHC	0.32398	3	0.9555
**Platelets**	**6.7173**	**3**	**0.08148**
WBC	4.917	3	0.178
ESR	5.7534	3	0.1242
**Fibrinogen**	**9.3893**	**3**	**0.02454**
CRP	3.1329	3	0.3716
**DAIS**	**48.708**	**3**	**<0.001**

**Table 5:** Results obtained from employing Pearson’s chi-squared test to establish the categorical variables that exhibit significant correlations with EDA.

Parameter	Chi-Square	df	*p*
Gender	2.9659	3	0.3969
**Calprotectin**	**26.828**	**6**	**<0.001**
Disease extent	8.1499	6	0.2273

**Table 6:** The performance metrics of the first MLP classifier (calprotectin excluded as a predictor).

	Train Set		Test Set	
	Actual Values		Actual Values	
Predicted Values	Remission	Activity	Remission	Activity
Remission	23	6	10	3
Activity	10	39	1	6
Accuracy	0.7949		0.8	
95% CI	(0.6884, 0.878)		(0.5634, 0.9427)	
*p* value	<0.001		0.01886	
Sensitivity	0.697		0.9091	
Specificity	0.8667		0.667	
PPV	0.7931		0.7692	
NPV	0.7959		0.8571	
AUC	0.86		0.9192	

**Table 7:** Performance metrics of the second MLP classifier (calprotectin included as a predictor).

	Train Set		Test Set	
	Actual Values		Actual Values	
Predicted Values	Remission	Activity	Remission	Activity
Remission	25	4	10	1
Activity	7	42	2	7
Accuracy	0.86		0.85	
95% CI	(0.7617, 0.9274)		(0.6211, 0.9679)	
*p* value	<0.001		0.016	
Sensitivity	0.7812		0.8333	
Specificity	0.9130		0.8750	
PPV	0.8621		0.9091	
NPV	0.8571		0.7778	
AUC	0.88		0.93	

## Data Availability

The data presented in this study are available on request from the corresponding author.
